# Molecular mechanisms of colistin- and multidrug-resistance in bacteria among patients with hospital-acquired infections

**DOI:** 10.2144/fsoa-2022-0055

**Published:** 2023-08-28

**Authors:** Surbhi Khurana, Amit Katiyar, Mamta Puraswani, Divya Sharma, Kamini Walia, Rajesh Malhotra, Purva Mathur

**Affiliations:** 1 Department of Laboratory Medicine, Jai Prakash Narayan Apex Trauma Centre, All India Institute of Medical Sciences, New Delhi; 2 Centralized Core Research Facility, Bioinformatics Facility, All India Institute of Medical Sciences, New Delhi, India; 3 Epidemiology & Communicable Diseases, Indian Council of Medical Research; 4 Department of Orthopedics, Chief, JPNA Trauma Centre, All India Institute of Medical Sciences, New Delhi

**Keywords:** antimicrobial resistance, colistin-resistant bacteria, hospital-acquired infections, multidrug resistance, whole genome sequencing

## Abstract

**Aim:**

The increasing burden of resistance in Gram-negative bacteria (GNB) is becoming a major issue for hospital-acquired infections. Therefore, understanding the molecular mechanisms is important.

**Methodology:**

Resistance genes of phenotypically colistin-resistant GNB (n = 60) were determined using whole genome sequencing. Antimicrobial susceptibility patterns were detected by Vitek®2 & broth microdilution.

**Results:**

Of these phenotypically colistin-resistant isolates, 78% were also genetically resistant to colistin. Activation of efflux pumps, and point-mutations in *pmrB*, and *MgrB* genes conferred colistin resistance among GNB. Eight different strains of *K. pneumoniae* were identified and ST43 was the most prominent strain with capsular type-specific (cps) gene KL30.

**Discussion:**

These results, in combination with rapid diagnostic methods, will help us better advice appropriate antimicrobial regimens.

The increasing antimicrobial resistance among bacteria causing hospital-associated infections (HAIs) is a threat to global health that increases morbidity and mortality among hospitalized patients. Gram-negative bacteria (GNB) are the most prominent causative agents of bacterial HAIs in such patients [1]. In the past few decades, bacteria acquired various resistance mechanisms to almost all available antibiotics including beta-lactams, fluoroquinolones, tetracycline and aminoglycosides [2]. Therefore, colistin and tigecycline remain effective antibiotics and have become the last resort of treatment for multidrug-resistant (MDR) GNB. In many countries, tigecycline has not been registered; leaving colistin as the only treatment option against multi-drug-resistant organisms. MDR organisms including, but not limited to, *Acinetobacter baumannii*, *Pseudomonas aeruginosa* and *Klebsiella pneumoniae* are developing a low level of resistance against colistin. Thus, GNB co-resistant to carbapenems, aminoglycosides, polymyxins and tigecycline (CAPT-resistance) are causing havoc [3–5]. Little is known about the development of a mechanism of resistance against colistin. Some bacteria, including *A. baumannii*, *P. aeruginosa*, and Enterobacteriaceae members like *Klebsiella* spp., *Enterobacter* spp. and *Escherichia coli* have acquired resistance against colistin. However, other bacteria, including *Serratia* spp., *Proteus* spp. and *Burkholderia* spp. are naturally resistant to this antibiotic. There are very limited data on acquired resistance to colistin. In gram-negative organisms such as *Salmonella enteric* and *P. aeruginosa*, modification of lipid A, a component of LPS, is considered to be the mechanism of colistin resistance. The addition of 4-amino-4-deoxy-l-arabinose (Ara4N) or/and phosphoethanolamine to lipid A removes the negative charge of lipid, lowering the affinity of positively charged colistin and thus contributing to the development of the colistin resistance [6]. Insertion of ISAba 11 into lipid biosynthesis genes like *lpxA* or *lpxC* resulted in the complete loss of LPS production and a high level of colistin resistance in *A. baumannii* [7]. Also, our finding suggest that, a mutation in the *PmrAB* two-component system like at least one amino acid change in *PmrB* or up-regulation of *pmrA* and *pmrB* resulted in colistin resistance. Modification of lipid A with the addition of phosphoethanolamine to lipid A mediated by the pmrAB two-component regulatory system also contributed to the colistin resistance [8]. In *Enterobacteriaceae*, changes in the regulatory loci *pmrA* and *phoP* are responsible for colistin resistance [9]. The *pmrA* locus encodes a two-component system, *PmrA–PmrB*, and a putative membrane protein *PmrC* [10,11]. The *phoP* locus encodes a distinct two-component system, *PhoP–PhoQ*, that governs resistance to several amphipathic antimicrobial peptides and was recently shown to also be required for resistance to colistin [12,13]. In KPC-producing *K. pneumoniae* (KPC-KP), alteration of the *mgrB* gene resulted in colistin resistance [14].

Although a major route of adaptive polymyxin resistance involves loss of lipopolysaccharide and the multi-component regulatory genes *PmrAB, PhoPQ, ParRS* and *CprRS*, the precise molecular details of these resistance mechanisms have also remained unclear.

Since colistin is the last resort of treatment for multidrug-resistant organisms, colistin resistance is a serious problem, especially in otherwise healthy trauma patients. Thus, we conducted the present study to identify the molecular mechanisms of colistin- and multidrug-resistance in GNB among trauma patients by whole genome sequencing.

## Methodology

### Type of study

This prospective observational study was conducted from January 2020 to March 2021.

### Sample collection

The clinical samples of trauma patients sent to the microbiology laboratory during the study duration who were resistant to colistin were included in the study.

The patients admitted in trauma centre are mainly accidental cases. Our microbiology laboratory receives different samples viz blood, urine, sputum, BAL samples, etc. for post-trauma microbiological investigations to advise antimicrobial regime & patient treatment. As a part of routine diagnostic tests these samples were processed as per standard microbiological procedures [18]. Blood samples were collected in BacT/ALERT aerobic blood culture bottles (bioMérieux, India), incubated, and monitored regularly using the BacT/ALERT system (bioMérieux, India). All bottles which signaled positive were removed from the instrument, Gram-stained and sub-cultured on blood agar and MacConkey agar. Urine samples were cultured on Cystine Lactose Electrolyte Deficient (CLED) agar. Phenotypic identification was confirmed with a Vitek^®^2 ID-GNB card (bioMérieux, India).

### Antimicrobial susceptibility testing

Antimicrobial susceptibility of the clinical isolates was determined both by disc diffusion method by the US Clinical and Laboratory Standards Institute [19,20] recommendations and the Vitek^®^2 system (using AST GN cards-AST YS08 and AST P628 Biomeriux, France). For the disc diffusion testing the following antimicrobials were tested: ceftazidime (30 μg), cefepime (30 μg), imipenem (10 μg), meropenem (10 μg), tigecycline (15 μg except for Pseudomonas Sp.) and piperacillin/tazobactam (100/10 μg), cefoperazone/sulbactam (75/30 μg), amikacin (30 μg), gentamicin (10 μg), ciprofloxacin (5 μg), levofloxacin (5 μg), aztreonam (30 μg), ticarcillin/clavulanic acid (75/10 μg), colistin (10 μg), trimethoprim-sulfamethoxazole (1.25/23.75 μg), Minocycline, Doripenem, amoxicillin/clavulanic acid (20/10 μg)). The interpretation of zone diameters was done as per CLSI guidelines [15,16]. The minimum inhibitory concentrations (MICs) were determined for all isolates by the automated Vitek^®^2 system (using AST GN cards-AST YS08 and AST P628 Biomeriux, France). The susceptibility of isolates to colistin was determined by the Broth microdilution (BMD) method. Briefly, sulfate salts of colistin (Sigma, St Louis, MO) were dissolved in cation-adjusted BBL Mueller Hinton II Broth (Becton Dickinson, NJ, USA). MIC dilutions ranging from 0.25 to 1,024 μg/ml were performed in untreated 96-well glass-coated microplates (Sigma, India). A MIC >2 μg/ml was considered resistant [17].

### Whole-genome sequencing

Genomic DNA was isolated from multiple sources including tissue, blood, respiratory samples, urine and water (environmental), and was quantified using a Qubit fluorometer (Life Technologies, USA). The quality and quantity of DNA were measured using a NanoDrop ND-1000 spectrophotometer (Thermo Fisher Scientific, MA, USA) and PFGE gel electrophoresis, respectively. The qualified DNA was cut into fragments by restriction enzymes. DNA fragments were end-repaired using deoxyadenosine bases before ligation with Illumina indexed adapters, amplified for 10 cycles of PCR, and sequenced employing v2 and v3 chemistry with paired-end 2 × 151 bp reads on NovaSeq 6000 (Illumina, USA). Output data files were de-multiplexed and transformed with Casava v.1.8.2. into FASTQ files (Illumina, Inc, USA).

### Genome assembly & annotation

The sequence quality was evaluated using FastQC0.11.4 and was pre-processed and assembled *de novo* with an A5-miseq pipeline [18,19]. Adapter and low-quality sequences (<Q30) were trimmed using trimmomatic v0.36 [20]. Read errors were corrected by SGA's k-mer-based error correction algorithm [21]. Paired-end reads data were assembled using DNA-UD algorithm and the subsequent quality of the assembled genome was evaluated by QUAST (http://quast.sourceforge.net/quast) [22]. The assembled bacterial genomes were aligned to reference genomes to nullify the risk of cross-contamination. The assembled contigs were annotated with Prokka (v1.12) genome annotation pipeline (http://www.vicbioinformatics.com/software.prokka.shtml) [23].

### Identification of resistance genes

The resistance genes in the assembled genomes were identified using the databases of antimicrobial resistance genes including cards Resistance Gene Identifier (RGI) software (RGI 4.2.0 and CARD 2.0.3 [24,25], NCBI AMR Finder Plus [26,27] and PathogenWatch v3.12.7 [28] AMRFinderPlus utilized NCBI Bacterial Antimicrobial Resistance Reference Gene Database (BioProject PRJNA313047) to find AMR-specific genes and proteins. RGI server was used to predict resistome(s) from nucleotide data based on homology and SNP models, where the ‘perfect and strict hits only’ criteria were chosen for the prediction. The ResFinder (webserver 3.0) was used to predict acquired genes and chromosomal point mutations mediating antimicrobial resistance in the DNA sequence of bacteria [29,30].

## Results

A total of 60 phenotypically colistin-resistant GNB isolates were analysed in this study. Of these, the most common isolates were identified as *K. pneumoniae* (n = 32), followed by *A. baumannii* (n = 17), *P. aeruginosa* (n = 6), *Enterobacter cloacae* (n = 3), *Escherichia coli* (n = 1) and *Klebsiella quasipneumoniae* (n = 1). The distribution of the source of samples of the pathogens is given in Table 1. The GNB were most frequently isolated from tissue (22/60, 37%).

**Table 1.** Distribution of source of samples and pathogens.

Source	*K. pneumoniae* (n = 33)	*A. baumannii* (n = 17)	*E. cloacae* (n = 3)	*P. aeruginosa* (n = 6)	*E. coli* (n = 1)
Tissue (n = 22)	10	8	1	2	1
Blood (n = 15)	10	3	2	0	0
Respiratory samples (n = 14)	8	3	0	3	0
Urine (n = 1)	0	0	0	1	0
Others^†^ (n = 7)	4	3	0	0	0
Water, environmental (n = 1)	1	0	0	0	0

† Others samples included fluid, bile and central venous pressure tip.

Antimicrobial susceptibility patterns were detected by WGS for resistance-determining genes (genotype) and by Vitek^®^2 (phenotype) for the following antibiotics: ticarcillin/clavulanic acid, piperacillin/tazobactam, ceftazidime, cefoperazone/sulbactam, cefepime, aztreonam, doripenem, imipenem, meropenem, amikacin, gentamicin, ciprofloxacin, levofloxacin, minocycline, tigecycline, colistin, trimethoprim/sulfamethoxazole, amoxicillin/clavulanic acid. The phenotypic antimicrobial resistance pattern of these pathogens against different antimicrobials tested is given in Table 2.

**Table 2.** Phenotypic antimicrobial resistance pattern of different pathogens.

	Ticarcillin/ clavulanic acid	Pipracillin/ tazobactam	Ceftazidime	Cefoperazone/ sulbactum	Cefepime	Aztreonam	Doripenem	Imipenem	Meropenem	Amikacin	Gentamicin	Ciprofloxacin	Levofloxacin	Minocycline	Tigecycline	Colistin	Trimethoprim/ sulfamethoxazole	Amoxicillin/ clavulanic acid
*K. pneumoniae* (n = 33)	33 (100%)	33 (100%)	33 (100%)	32 (97%)	32 (97%)	33 (100%)	30 (91%)	19 (58%)	32 (97%)	29 (88%)	27 (82%)	33 (100%)	33 (100%)	14 (42%)	2 (6%)	32 (97%)	23 (69%)	30 (91%)
*A. baumannii* (n = 17)	17 (100%)	16 (94%)	17 (100%)	6 (35%)	16 (94%)	NA	17 (100%)	16 (94%)	16 (94%)	3 (18)	14 (82%)	16 (94%)	17 (100%)	15 (88%)	1 (6%)	17 (100%)	16 (94%)	NA
*P. aeruginosa* (n = 6)	5 (83%)	5 (83%)	5 (83%)	5 (83%)	5 (83%)	4 (67%)	5 (83%)	5 (83%)	5 (83%)	5 (83%)	5 (83%)	5 (83%)	5 (83%)	NA	6 (100%)	6 (100%)	NA	NA
*E. cloacae* (n = 3)	0	0	0	0	0	0	0	0	0	0	0	0	0	0	0	3 (100%)	1 (33%)	3 (100%)
*E. coli* (n = 1)	1 (100%)	0	1 (100%)	0	1 (100%)	1 (100%)	0	0	0	0	1 (100%)	1 (100%)	1 (100%)	0	0	1 (100%)	1 (100%)	NA

The WGS analyses revealed 8 different strains of *K. pneumoniae*; ST43 was the most prominent strain with capsular type-specific (cps) gene KL30. The frequency of different strains and capsular genes were shown in Table 3.

**Table 3.** Frequency of different strains and capsular specific type genes.

Strain	n (%)	Gene	n (%)
ST43	14 (44%)	*KL30*	15 (47%)
ST15	7 (29%)	*KL64*	6 (19%)
ST395	4 (13%)	*KL112*	5 (16%)
ST2096	2 (6%)	*KL2*	2 (6%)
ST231	2 (6%)	*KL51*	2 (6%)
ST16	1 (3%)	*KL36*	1 (3%)
ST43-ILV	1 (3%)	*KL81*	1 (3%)
ST437	1 (3%)		

WGS analyses revealed that 47/60 isolates were genotypically colistin-resistant; *K. pneumoniae* (n = 33/33), *A. baumannii* (n = 6/17), *E. cloacae* (n = 3/3) and *P. aeruginosa* (n = 5/6). Table 4 details the sensitivity and specificity of genotype versus phenotype resistance pattern among *K. pneumoniae.* We also evaluated genotypic and phenotypic correlation and found that most of the isolates showed resistance both genetically and phenotypically except for Amikacin and Trimethoprim/sulfamethoxazole. Table 5 details the colistin resistance isolates and genes encoding resistance. We evaluated for correlation and did not find any correlation between the resistance gene and capsule type (r = 0.056, p = 0.203); capsule and colistin (r = 0.75, p = 0.13).

**Table 4.** The sensitivity and specificity of genotype versus phenotype resistance pattern among *K. pneumoniae*.

Antibiotic	Phenotypically non-susceptible		Phenotypically susceptible		Sensitivity (%)	Specificity (%)	p-value
	Genotypically non-susceptible	Genotypically susceptible	Genotypically non-susceptible	Genotypically susceptible			
Amikacin	28	1	2	1	97	33	0.04
Ciprofloxacin	33	0	0	0	100	100	0
Trimethoprim/sulfamethoxazole	17	6	3	7	74	70	0.018
Cefepime	32	0	0	1	100	100	0
Imipenem	32	0	0	1	100	100	0
Piperacillin/tazobactam	33	0	0	0	100	100	0
Colistin	33	0	0	0	100	100	0

**Table 5.** Details of colistin resistance isolates and genes encoding resistance.

Colistin resistance gene	Strains
*KpnE, KpnF, KpnG, KpnH*	Kp1-Kp19, Kp22-Kp29, Kp31-Kp33
*KpnE, KpnF*	Kp20, Kp21, Eb1-Eb3, Pa4, Ab8
*KpnF, KpnG, KpnH*	Kp30
*KpnE, KpnF, KpnH*	Ab1
*KpnF, KpnH*	Ab3, Ab5
*CpxR, KpnH*	Pa1
*CpxR*	Pa2, Pa5, Pa6
*KpnH*	Ab2, Ab4
*pmrB_R256G*	Kp14, Kp19, Kp22
*pmrB_T157P*	Kp9-Kp13, Kp18, Kp24

Ab: *A. baumanni*; Eb: *E. cloacae*; Kp: *K. pneumoniae*; Pa: *P. aeruginosa*.

Most of the isolates were resistant to colistin may be due to the presence of genes accountable for the activation of efflux pumps. 6 isolates of *A. baumanni* (Ab1-Ab5 and Ab8), 3 isolates of *E. cloacae* (Eb1, Eb2, and Eb3), 5 isolates of *P. aeruginosa* (Pa1-Pa3, Pa5, and Pa6) were positive for activation of efflux pumps genes (72 genes) and showed resistant to colistin. These genes belong to families of ATP-binding cassette (ABC) antibiotic efflux pump, major facilitator superfamily (MFS), resistance nodulation cell division, and small multidrug resistance (SMR). Most of the genes belong to gene family RND antibiotic efflux pump. Among *K. pneumoniae*, all isolates harbored activated efflux pump genes (*AdeF, BaeR, Ccl5, EmrR, H-NS, KpnE, KpnF, KpnG, KpnH, MarA, MsbA, OqxA, OqxB, tet(A), tet(B)*, *tet(D)*); 13 isolates had a mutation in the *pmrB* gene and 6 isolates had a mutation in the *MgrB* gene in combination with activated efflux pumps. Three isolates had a mutation in both the *pmrB* and *MgrB* genes in combination with activated efflux pumps. Twelve strains showed point mutation in the *pmrB* gene. Out of these 5 strains (Kp4, Kp5, Kp14, Kp19, and Kp22) had* pmrB* R256-G mutation; 7 strains (Kp9-Kp13, Kp18, and Kp24) had *pmrB* T157-P mutation.

Table 6 details the genes encoding resistance to various classes of antimicrobials, which were identified in the study, as follows:

**Table 6.** Genes encoding for resistance to different classes of antimicrobials.

Antibiotic class	Organism (n)	Genes encoding resistance
Aminoglycoside	*K. pneumoniae* (n = 33)	*aac(3)-Iid, aadA1, aadA2, rmtB, aac(6′)-Ib-Hangzhou, rmtF, aac(3)-IId, aph(3″)-Ib, aph(6)-Id, rmtC, aac(6′)-Ib3, aac(6′)-Ib-cr, armA, rmtF, aph(3′)-Ia, armA, aac(6′)-Ib-cr, aac(3)-IIa*
	*A. baumanni* (n = 14)	*aac(6′)-Ic, aph(3″)-Ib, aph(3′)-Ia, aph(3′)-VI, aph(3′)-Via, aph(6)-Id, armA*
	*E. coli* (n = 2)	*aph(3″)-Ib, aph(3′)-Ia, aph(6)-Id, armA, aac(6′)-Ib3, aadA1*
	*E. cloacae* (n = 2)	*aph(3″)-Ib, aph(3′)-Ilb, aac(6′)-Il, aph(6)-Id, aadA1, ant(2″)-Ia, aph(3′)-VI*
	*P. aeruginosa* (n = 5)	*aac(3)-Ic, aac(6′)-Ib3, aadA5, aadA1, armA, rmtB, aph(6)-Id, aph(3′)-Ia, aph(3′)-Ib, aph(3′)-Ilb, aph(3′)-Via, aac(6′)-Ib-Hangzhou*
eta-lactam	*K. pneumoniae* (n = 33)	*blaCTX-M-15, blaNDM-5, blaOXA-181, blaSHV-11, blaSHV-13, blaSHV-145, blaSHV-179, blaSHV-185, blaSHV-194, blaSHV-199, blaSHV-26, blaSHV-31, blaSHV-60, blaSHV-61, blaSHV-70, blaSHV-78, blaSHV-98, blaSHV-40, blaSHV-56, blaSHV-79, blaSHV-85, blaSHV-89, blaTEM-1B, blaSHV-12, blaOXA-232, blaCMY-6, blaNDM-1, blaSHV-106, blaSHV-28, blaOXA-1, blaSHV-182, blaSHV-55, blaOXA-23, blaTEM-1A, blaSHV-40, blaTEM-1C, blaNDM-4, blaOXA-9*
	*A. baumannii* (n = 16)	*blaADC-25, blaCMH-3, blaDHA-1, blaNDM-1, blaOXA-23, blaOXA-232, blaOXA-275, blaOXA-66, blaSRT-2, blaSST-1, blaTEM-104, blaTEM-1B, blaTEM-1C, blaDHA-17*
	*Escherichia coli* (n = 2)	*blaADC-25, blaNDM-1, blaOXA-23, blaOXA-66, blaTEM-1D*
	*E. cloacae* (n = 3)	*blaCMH-3, blaCTX-M-15, blaNDM-5*
	*P. aeruginosa* (n = 6)	*blaADC-25, blaKPC-2, blaDHA-22, blaOXA-1, blaOXA-23, blaOXA-301, blaOXA-320, blaOXA-396, blaOXA-66, blaOXA-488, blaOXA-494, blaOXA-534, blaPAO, blaVEB-5, blaVIM-2*
Quinolone	*K. pneumoniae* (n = 31)	*oqxA, oqxB, qnrS1, aac(6′)-Ib-cr, qnrB1*
	*A. baumanni* (n = 1)	*qnrB4*
	*E. coli* (n = 1)	*aac(6′)-Ib-cr*
	*E. cloacae* (n = 3)	*qnrS1, crpP, qnrVC1*
	*P. aeruginosa* (n = 4)	*aac(6′)-Ib-cr, crpP*
Sulphonamide	*K. pneumoniae* (n = 20)	*sul1*
	*A. baumanni* (n = 6)	*sul1, sul2*
	*E. coli* (n = 2)	*sul1, sul2*
	*E. cloacae* (n = 2)	*sul1, sul2*
	*P. aeruginosa* (n = 3)	*sul1*

### β-lactams resistance genes

All 33 isolates of *K. pneumoniae* harbored β-lactams resistant genes. Different resistant genes found were: *bla-CMY-6, bla-OXA-1, bla-SHV-28, bla-TEM-1D, bla-OXA-9* and *bla-SHV-187*. Among these 33 isolates 42% (14/33) harbored single resistant gene *bla-TEM-1D*, 16/33 carried in combination with other genes like *bla-CMY-6, bla-OXA-1, bla-OXA-9, bla-SHV-28, bla-SHV-187. Bla-SHV-28 and bla-OXA-1* resistant gene was harbored by a single isolate individually.

### β-lactams + inhibitor resistance genes

19 isolates of *K. pneumoniae* harbored β-lactams + inhibitor *bla-SHV-11* resistant gene.

### ESBL resistance genes

All 32 isolates harbored ESBL resistant *bla-CTX-M-*15 gene.

### Carbapenem-resistance genes

Twelve isolates of *K. pneumoniae* harbored the carbapenem-resistant gene *OXA-181*. Seven isolates harbored the *OXA-232* carbapenem-resistant gene. Twelve isolates had the combination of *NDM* and *OXA* in different allelic forms. The single isolate was resistant due to the presence of the *NDM-1* gene alone.

### Sulfonamide resistance gene

Nineteen isolates harbored the sulphonamide resistance gene; all attributed to the presence of* Sul* gene.

## Discussion

A major cause of concern is the increasing number of reports of CAPT resistance across the globe [31]. Moreover, there is a scarcity of clinical data to guide the treatment of MDR-GNB, and are based exclusively on small case series and a few case reports. To aim for timely and targeted treatment of patients with infections by such MDR GNB, a better understanding of resistance mechanisms is essential. Although at present, a synergistic combination of older agents for example- fosfomycin- or polymyxin-based synergistic combinations, may be the last resort option, their use against CAPT-resistant GNB requires further investigation [32].

Our study focused on the most problematic species, namely *K. pneumoniae, P. aeruginosa* and *A. baumannii* among others. WGS analyses showed that only 78% of the phenotypically colistin-resistant isolates were genotypically colistin-resistant. Thus, the phenotypic tests probably do not reflect the actual resistance burden. Previous studies conducted at our center also highlighted the possibilities of a complex combination of mutations that led to colistin resistance [33,34]. Activation of efflux pumps, and point-mutations in *pmrB*, and *MgrB* genes conferred colistin resistance among these GNB. In addition to genotypic analyses of genes conferring colistin resistance in these isolates, we studied the genes encoding the most predominant resistance enzymes among these MDR-GNB viz. β-lactams resistance genes, β-lactams + inhibitor resistance genes, ESBL resistance genes, carbapenem-resistance genes and sulfonamide resistance gene. As the previous studies reported a high proportion of ESBL production from the isolates of middle east Asia [35], this study also found a high proportion of isolates were positive for ESBL*.* Hence, continuous and large-scale surveillance of molecular mechanisms is required and emphasis should be laid on culture-based and synergistic antimicrobial treatment regimes.

The limitation of our study remains the limited number of isolates studied at a single centre. More such data from different geographies would help gain more insights. Until new drugs become widely available in clinical practice, more research encompassing the study of molecular epidemiological mechanisms of resistance, pharmacokinetics & pharmacodynamics, and outcome studies is crucial. This understanding becomes particularly advantageous if there is access to accurate & rapid laboratory methods that can determine the molecular mechanisms of resistance. Many such methods are available, including lower-cost phenotypical assays which are suitable for microbiology laboratories of any capacity. However, PCR tests and high-throughput diagnostic tests would not only be expensive but also require expert and trained personnel to interpret such data. These data will guide the selection of appropriate antimicrobials for the management of MDR GNB, especially the CAPT-resistant GNB.

## Conclusion

This study found *K. pneumoniae* to be the commonest organism with ST43 being the most prominent strain encoding capsular type-specific (cps) gene KL30. All the organisms contained several antimicrobial resistant genes, particularly genes for antibiotic efflux pumps, *pmrB* and *MgrB*. It appears that these complementary mutations conferred colistin resistance in these organisms. Since colistin is the ‘last resort’ antimicrobial, uniform surveillance of molecular mechanisms would enable adoption of the synergistic antimicrobial treatment regimes.

**Summary points**

- In this study, we found that 78% of the phenotypically colistin-resistant isolates were genotypically colistin resistant.
- Thus, the phenotypic tests probably do not reflect the actual resistance burden.
- Activation of efflux pumps, and point-mutations in *pmrB*, and *MgrB* genes conferred colistin resistance among these Gram-negative bacteria (GNB).
- We also elucidated the genes encoding the most predominant resistance enzymes among these MDR-GNB viz. β-lactams resistance genes, β-lactams + inhibitor resistance genes, ESBL resistance genes, carbapenem-resistance genes, and Sulfonamide resistance genes.
